# Salivary Gland Tumors in Maxillofacial Region: A Retrospective Study of 130 Cases in a Southern Iranian Population

**DOI:** 10.4061/2011/934350

**Published:** 2011-07-03

**Authors:** Mahmoud Shishegar, Mohamad J. Ashraf, Negar Azarpira, Bijan Khademi, Basir Hashemi, Amir Ashrafi

**Affiliations:** ^1^Departments of Otolaryngology and Maxillofacial Surgery, Shiraz University of Medical Sciences, Shiraz, Iran; ^2^Department of Pathology, Shiraz University of Medical Sciences, Shiraz, Iran; ^3^Organ Transplant Research Center, Nemazi Hospital, Shiraz University of Medical Sciences, P.O. Box 71935-1119, Shiraz, Iran

## Abstract

Tumors of the salivary glands are uncommon head and neck neoplasia. We conducted a retrospective study of 392 cases over the last 6 years in Shiraz, south of Iran, to investigate the clinicopathological features of these tumors in Iranian population. The age of the patients ranged from 8 to 85 years, with the mean age 44.57 ± 14.65 years and male-to-female (M : F) ratio was 1.02 : 1. For benign tumors, there was a propensity towards females, whereas the malignant tumor was more common in males. The ratio of benign tumors to malignancies was 2.19 : 1. Pleomorphic adenoma (PA) was the most common tumor and accounted for 85% of all benign tumors, followed by Warthin's tumor (8.6%). Of the 125 malignancies, adenoid cystic carcinoma (40%), mucoepidermoid carcinoma (24%) and invasive squamous cell carcinoma (16%) were the most common histological types. Most of the salivary gland tumors (75%) originated from major salivary glands and the remained (25%) originated from minor glands. The parotid gland was the most common site both in benign and malignant tumors. Most of our findings were similar to those in the literature, with some variations. The salivary tumors slightly predominated in males. Adenoid cystic carcinoma and mucoepidermoid carcinoma constituted the most common malignancies.

## 1. Introduction

Salivary gland tissues are diffusely distributed in the upper aerodigestive tract. The parotid, submandibular, and sublingual glands are the major salivary glands. Minor salivary glands are present in many sites, such as the lips, gingiva, cheek, palate, tongue, oropharynx, paranasal sinuses, and parapharyngeal space. Salivary gland tumors are relatively uncommon lesions accounting for 3–6% of all head and neck neoplasms [1]. The global incidence of these tumors is 0.4–13.5 per 100,000 persons annually [2–4]. These neoplasms composed heterogeneous groups of tumors with variable histological pictures. The site, patient age, and sex distributions of different types of salivary gland neoplasms vary with race and geographic location. The incidence of these tumors is different in between geographic areas and ethnic groups [2, 3, 5]. 

In the English literature, there is little report [1] on salivary gland tumors in Iranian population. The aim of this study was to analyze the relative frequency, location, patient sex, and age of salivary gland tumors in the southern Iranian population over the last 6 years.

## 2. Material and Methods

This study included patients with primary epithelial salivary gland neoplasms between 2004 to 2009, who underwent operations in the Department of Maxillofacial Surgery, Khalili Hospital, Shiraz. Hematoxylin-eosin- (H&E-) stained slides of all cases were reviewed by two pathologists based on the 2005 World Health Organization classification of head and neck tumors criteria [6]. Information regarding age, gender, and anatomical location of the tumors was collected from the patients' hospital records. This research was approved by the Ethics Committee of Shiraz University of Medical Sciences. The data were analyzed for their distribution of patient's sex and age and anatomical location of tumors.

## 3. Results

### 3.1. Histological Types

**Figure 1:**
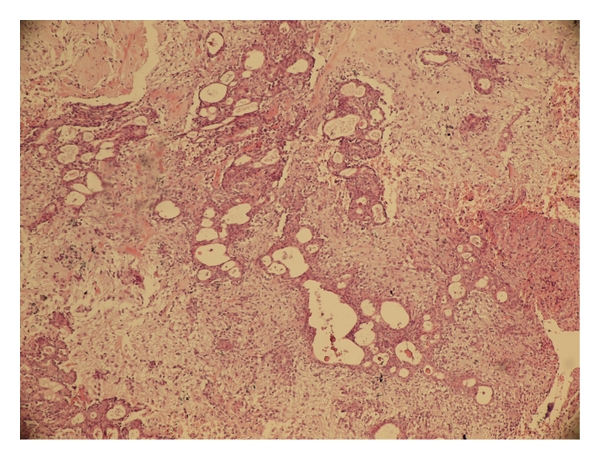
Benign mixed tumor (pleomorphic adenoma) with a biphasic admixture of epithelium and stroma (H&E ×200).

392 patients underwent operations for salivary gland tumors during this period. Among them, 267 (68.2%) were benign and 125 (31.8%) were malignant. The ratio of benign tumors to malignancies was 2.19 : 1. The distribution of histological patterns by anatomical locations for benign and malignant salivary tumors is shown in Table 1 and Figure 1, respectively.

Pleomorphic adenoma (PA) was the most common tumor and accounted for 85% (227/267) of all benign tumors (Figure 1), followed by Warthin's tumor (23/267, 8.6%). Myoepithelioma, basal-cell adenoma, and oncocytoma accounted for 4.5% (12/267), 1.1% (3/267) and 0.7% (2/267) of benign tumors, respectively (Table 1).

**Figure 2:**
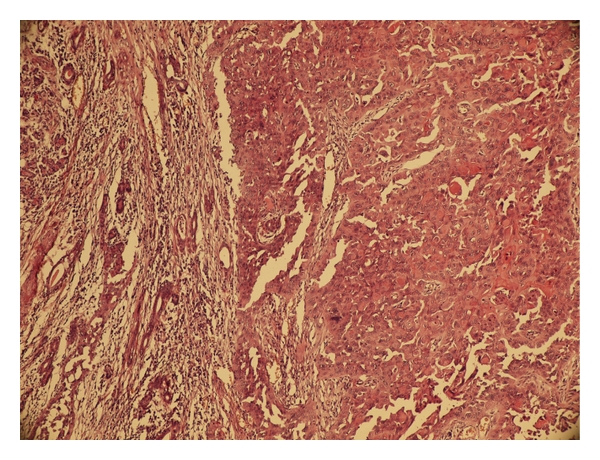
High-grade mucoepidermoid carcinoma composed of squamous with few intermediate and clear cells (H&E ×200).

**Figure 3:**
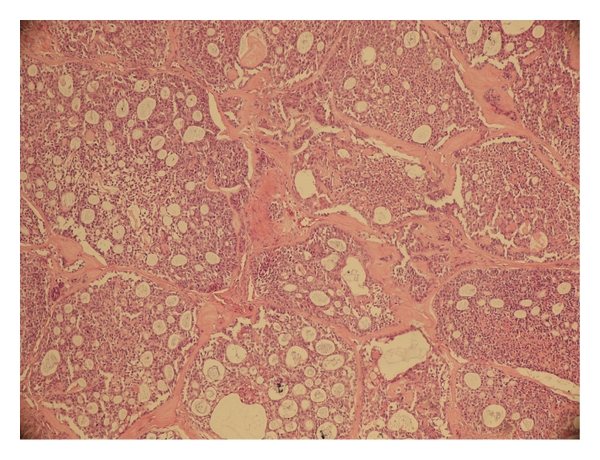
Adenoid cystic carcinoma, nests of cells of rather bland appearance are arranged concentrically around gland-like spaces (H&E ×200).

Of the 125 malignancies, adenoid cystic carcinoma (ACC, 50/125, 40%) (Figure 2), mucoepidermoid carcinoma (MEC, 30/125, 24%), (Figure 3), and squamous cell carcinoma (SCC, 20/125, 16%) were the most common histological types, followed by acinic cell carcinoma (6/125, 4.8%), adenocarcinoma, not otherwise specified (NOS) (5/125, 4%) and epithelial-myoepithelial carcinoma (4/125, 3%). Carcinoma ex-pleomorphic adenoma (2/125, 1%), salivary duct carcinomas (2/125, 1%), polymorphous low-grade adenocarcinoma (2/125, 1%) and basal-cell adenocarcinoma (2/125, 1%) were rare tumors. Twenty-two cases of squamous cell carcinoma (22/125, 17%) was reported in this series that all of them were direct invasion from overlying skin or metastatic to intraparotid lymph nodes (Table 1).

### 3.2. Locations

Most of the salivary gland tumors (297/392, 75%) originated from major salivary glands and the remained (95/392, 25%) originated from minor glands mainly located in the palate and lips. The parotid gland was the most common site both in benign (175/297, 59%) and malignant (56/125, 45%) tumors (Figure 2). Most of the tumors in the minor salivary glands were malignant rather than benign (53/34) and the palate was the most frequent location for minor gland tumors. Among benign tumors, almost all Warthin's tumors (100%), oncocytomas (100%), basal-cell adenomas (100%), and most myoepitheliomas (60%) were located in the parotid gland. 

Among the malignancies, ACC and MEC were the most common types. ACC more frequently occurred in minor salivary glands, but the MEC was more cited in major salivary glands. Neither malignant nor benign tumors possessed a dominance of left- or right-side involvement. Bilateral involvement was not present in this study.

### 3.3. Age and Sex

Among 392 patients with salivary gland tumors, 198 were male, and 194 were female; the male-to-female (M : F) ratio was 1.02 : 1. For benign tumors, there was a propensity towards females (130/137, M : F = 0.9 : 1), whereas the malignant tumors were more common in males (68/57, M : F = 1.1 : 1), (Figure 2). 

The age of the patients in this study ranged from 8 to 85 years, with the mean ± SD = 44.57 ± 14.65 years. The peak incidence for both benign and malignant salivary gland tumors was the fifth decade of life (Figure 3). 

The peak incidence of PA is the fourth to sixth decades. Warthin's tumor is more prevalent in the 5-6th decade, and the oncocytoma and basal-cell adenoma are more common in fourth decade of life. For malignant tumors, the highest incidences of MEC and ACC were all in the fifth to sixth decade of life.

The total number of tumors (benign and malignant) occurring in young people under 20 years was 31, representing 8% of all tumors. In this age group, benign tumors were predominant (Figure 3), and PA was the most common type of tumor (23/31, 74%), followed by ACC (4/31, 12%). The ACC was the most common type of malignant tumors in this age group.

## 4. Discussion

Khalili Hospital is the largest referral hospital for maxillofacial tumors in the south of Iran, and many salivary gland tumors are treated in this hospital. In this study, benign salivary gland comprised 68% of all salivary tumors and predominated in major glands, similar to the rates reported by authors in the west of Iran, China, Jordan, UK, USA, India, Brazil, Nigeria, Congo, Uganda, Bratislava, and Sri Lanka [1, 2, 4, 7, 9–12, 14] (Table 2). In all these reports from different countries, benign tumors accounted for more than 50% of all salivary tumors, suggesting that benign tumors are predominant in salivary gland tumors worldwide.

In this study, PA was the most common type of salivary gland tumor (58%). This was consistent with other reports from different parts of the world, which have considered prevalence rates for PA between 40–65% (Table 2). The majority of PA was in major salivary glands. This finding was similar to a WHO report [6], in which approximately 80% of all PAs were occurred in the parotid gland, and 10% developed in the various minor glands.

The second most common benign tumor in this study, Warthin's tumor, comprised 23% of all salivary tumors, which was less than the prevalence in Denmark and parts of Pennsylvania (about 30% of parotid tumors) [17, 18]. This tumor was rare in African populations [11]. Most of these tumors occurred in males (78%), and the M : F ratio was 3.6 : 1. Previous studies mentioned an increasing incidence of Warthin's tumor in females during the past 50 years, and the M : F ratio changed from 10 : 1 to 1.2 : 1, which may be related to the increased numbers of female smokers [6, 17]. In this study the majority of patients with Warthin's tumor had a history of tobacco smoking.

The reported frequencies for malignant salivary gland ranged were between 10–46%, and the MEC was the most common malignant tumor, with a prevalence ranging from 4–12% [6] (Table 2). Our data showed that malignancies comprised 32% of all salivary gland, and the ACC was the most common one (13%). The overall incidence of malignant tumors was similar to those reported from west of Iran and other countries [1, 2, 4, 7, 9–12, 14]. The higher prevalence of ACC was near the reported incidence from China and Congo [4, 9], but in the west of Iran, China, Jordan, UK, USA, India, Brazil, Nigeria, Uganda, and Bratislava the frequency of MEC was higher than that of ACC, and MEC was the most common malignancy (Table 2). These findings suggest a geographic variation in the frequencies of ACC and MEC. However, our report was different from Buchner et al. findings. They studied relative frequency of intraoral minor salivary gland tumors in northern California, USA. MEC was the most common (21.8%), followed by PLGA (7.1%) and ACC (6.3%) [19]. According to WHO classification of salivary gland tumors 2005, PLGA is the second more common malignant tumor of minor salivary gland, being surpassed only by MEC.

Most studies [6, 7, 10] revealed that the occurrence of salivary gland tumors was slightly higher in females. In the present study, males were slightly more affected (M : F = 1.02 : 1) like the finding in previous study [4]. The reason for this was the significant male predominance for Warthin's tumor and SCC.

In summary, this study was an epidemiological analysis of salivary gland tumors in the south Iranian population. Most of the findings about the distribution of histological type, age, and sex in this population were similar to those reported in the literature. However, there were few racial and geographic variations in the frequency and distribution of tumors between this study and other populations. PA was the most common benign and ACC ranked as the most common malignant salivary gland tumors followed by MEC. The overall occurrence of salivary gland tumors was slightly higher in males.

The reason for these differences remains unclear. Therefore, more research on this field is greatly encouraged.

## Figures and Tables

**Table 1:** Location and histological types of benign and malignant salivary glands tumors.

Tumor type	Number (%)	Major salivary gland (*n* = 297)	Minor salivary gland (*n* = 95)
Pleomorphic adenoma	227 (58)	188	39
Warthins' tumor	23 (6)	23	0
Myoepithelioma	12 (3)	7	5
Basal-cell adenoma	3 (0.7)	3	0
Oncocytoma	2 (0.6)	2	0
Mucoepidermoid carcinoma	30 (8)	26	4
Adenoid cystic carcinoma	50 (13)	14	36
Adenocarcinoma NOS	5 (1)	0	5
Acinic cell carcinoma	6 (1.5)	5	1
carcinoma ex-pleomorphic adenoma	2 (0.6)	0	2
Epithelial-myoepithelial carcinoma	4 (1)	4	0
salivary duct carcinoma	2 (0.6)	2	0
Polymorphous low-grade adenocarcinoma	2 (0.6)	1	1
Basal-cell adenocarcinoma	2 (0.6)	0	2
Squamous cell carcinoma (Invasion from overlying skin)	22 (5)	22	0

**Table 2:** Comparison of reported distribution of salivary gland tumors in south of Iran and other countries.

	Total	Benign	Malignant	PA	ACC	MEC	Reference
Current study	392	267 (68)	124 (32)	227 (58)	50 (13)	30 (8)	—
Iran, west	130	89 (68)	41 (32)	85 (65)	3 (2)	15 (11)	[1]
China	6982	4743 (68)	2239 (32)	3281 (47)	681 (10)	673 (10)	[4]
UK	741	481 (65)	260 (35)	329 (44)	62 (8)	85 (11)	[7]
Italy	454	405 (89)	49 (11)	287 (63)	8 (2)	15 (3)	[8]
Jordan	221	151 (68)	70 (32)	139 (63)	12 (5)	38 (17)	[2]
Congo	275	180 (65)	95 (35)	152 (55)	44 (16)	22 (8)	[9]
Brazil	496	335 (68)	161 (32)	269 (54)	39 (8)	67 (14)	[10]
Uganda	268	145 (54)	123 (46)	107 (40)	36 (13)	25 (9)	[11]
USA	218	198 (90)	20 (9)	137 (62)	0 (0)	20 (9)	[12]
Bratislava	767	649 (85)	118 (15)	550 (71)	65 (8)	53 (6)	[13]
Nigeria	78	44 (56)	34 (43)	37 (49)	1 (1)	18 (23)	[14]
Sri Lanka	713	356 (50)	356 (50)	274 (38)	96 (13)	154 (22)	[15]
India	684	422 (62)	262 (38)	588 (86)	123 (18)	171 (25)	[16]
